# Case report: Non-response to fluoxetine in a homozygous 5-HTTLPR S-allele carrier of the serotonin transporter gene

**DOI:** 10.3389/fpsyt.2022.942268

**Published:** 2022-07-15

**Authors:** Céline K. Stäuble, Rebecca Meier, Markus L. Lampert, Thorsten Mikoteit, Martin Hatzinger, Samuel S. Allemann, Kurt E. Hersberger, Henriette E. Meyer zu Schwabedissen

**Affiliations:** ^1^Biopharmacy, Department of Pharmaceutical Sciences, University of Basel, Basel, Switzerland; ^2^Pharmaceutical Care, Department of Pharmaceutical Sciences, University of Basel, Basel, Switzerland; ^3^Institute of Hospital Pharmacy, Solothurner Spitäler AG, Olten, Switzerland; ^4^Psychiatric Services Solothurn, Solothurner Spitälerler AG and Faculty of Medicine, University of Basel, Solothurn, Switzerland

**Keywords:** pharmacogenetics, depression, pharmaceutical care, *SLC6A4*, 5-HTT, *ABCB1*, pharmacodynamics, venlafaxine

## Abstract

We report the case of a 50-year-old male with major depressive disorder (MDD) to illustrate the challenge of finding effective antidepressant pharmacotherapy and the role that the patient’s genetic makeup may play. Recent treatment attempts before clinic admission included venlafaxine and fluoxetine. Venlafaxine was discontinued due to lack of response, and subsequently switched to fluoxetine based on pharmacogenotyping of the P-glycoprotein transporter (P-gp, encoded by *ABCB1*) by the outpatient psychiatrist. Despite steady state serum levels within the therapeutic range, the patient did not benefit from fluoxetine either, necessitating admission to our clinic. Here a clinical pharmacist-led medication review including additional pharmacogenetic (PGx) analysis resulted in the change of the antidepressant therapy to bupropion. Under the new regimen, established in the in-patient-setting, the patient remitted. However, based on the assessed pharmacokinetics-related gene variants, including *CYP*s and *ABCB1*, non-response to fluoxetine could not be conclusively explained. Therefore, we retrospectively selected the serotonin transporter (SERT1, encoded by *SLC6A4*) for further genetic analysis of pharmacodynamic variability. The patient presented to be a homozygous carrier of the short allele variant in the 5-HTTLPR (S/S) located within the *SLC6A4* promoter region, which has been associated with a reduced expression of the SERT1. This case points out the potential relevance of panel PGx testing considering polymorphisms in genes of pharmacokinetic as well as pharmacodynamic relevance.

## Introduction

Major depressive disorder (MDD) is a common condition that imposes a high disease burden on the individual patient (1). However, not only the affected patients, but also the healthcare system and society are challenged by the disorder, in particular due to the resulting costs. The majority of the costs are of indirect kind and arise due to unemployment, sick leave and early retirement (2, 3). Therefore, it is important to effectively treat MDD. A relevant pillar in the treatment of MDD is pharmacotherapy. Fortunately, a wide range of marketed antidepressants is available today for clinicians and patients to choose from. Still, treatment of MDD remains challenging as it is known that up to 50% of unipolar depressed patients treated with antidepressants do not respond to their first-line treatment (4, 5). Ineffective antidepressant treatment may prolong the disease state, increasing the burden on the patient, the health care system, and society.

Multiple factors impact the response to antidepressants, including the patient’s genetic makeup. On the one hand, genetic variation can alter the expression and/or activity of enzymes and transporters involved in drug absorption, distribution, metabolism, or excretion (ADME), causing interindividual differences in pharmacokinetics. On the other hand, genetic variation can affect the expression and/or structure of drug targets, potentially interfering with pharmacodynamics. Pharmacokinetic as well as pharmacodynamic alterations may impact both, tolerability and effectiveness of a drug (6).

The role of genetic predisposition in antidepressant response is extensively discussed in basic research as well as in clinical practice (7–9). So far, mainly pharmacokinetics-related genetic markers have found their way into clinical practice. In particular, compelling evidence on the impact of genetic variation of the enzyme cytochrome P450 (CYP) 2D6 and CYP2C19 has led to the publication of guidelines with recommendations for genotype-based selection and dosing of selective serotonin reuptake inhibitors (SSRI) and tricyclic antidepressants (10, 11). Both cytochromes, CYP2D6 and CYP2C19, are highly polymorphic which is reflected by the fact that over 60% of the general European population have a predicted phenotype that deviates from a normal metabolizer (extensive metabolizer, EM) (12). Moreover, the Swiss Society for Anxiety and Depression (SGAD) recommends genotyping of the P-glycoprotein (P-gp, encoded by *ABCB1*) after antidepressant treatment failure (13). P-gp is an efflux transporter which is also expressed in the blood-brain barrier (BBB), where it has an important gatekeeping role and extrudes various substances including certain antidepressants (14). It is hypothesized that carriers of the respective reference variant (wildtype) have restricted permeability of their BBB to antidepressants that are P-gp-substrates and therefore may only reach a limited concentration in the brain at their site of action (15). This theory is based on a limited number of clinical studies that associated certain *ABCB1* polymorphisms to antidepressant treatment response (15–17).

In addition to the afore described pharmacokinetics-related genetic variants, there is also evidence indicating effects of polymorphisms in pharmacodynamic-related genes on antidepressant efficacy and tolerability (18). It still remains controversial whether genetic variants in pharmacodynamically relevant antidepressant targets should be adopted in clinical practice. To date, there are no treatment recommendations based on any pharmacodynamic-related gene variants available for antidepressants. Extensive research is ongoing in this area, in particular studies on polymorphisms in the *SLC6A4* gene, encoding for the serotonin transporter (SERT1). The promoter region of the *SLC6A4* harbors a highly polymorphic region, named 5-HTTLPR (rs774676466), with a 44 base pair insertion-deletion (INDEL) variation (19). The short variant (S-allele) has a minor allele frequency of about 20% on a global average (20) and has been linked with reduced transcriptional activity and therefore limited expression of the encoded SERT1 (19). The SERT1 facilitates the reuptake of serotonin from the synaptic cleft into the presynapse and is a relevant target of various antidepressants, especially SSRIs (21). Hitherto, multiple studies linked the 5-HTTLPR variation with antidepressant therapy outcome (22, 23). However, it is difficult to apply these findings in practice, as there are currently no guidelines available associating *SLC6A4* genotypes with concrete recommendations for antidepressant selection and dosing. Herein we are reporting a case, where the *SLC6A4* 5-HTTLPR variation was likely causative in the tediously protracted search for an effective antidepressant.

## Case presentation

### Clinical case and medication history

A 50-year-old male with a long lasting history of recurrent MDD (ICD-10 F33), admitted himself to the medical emergency ward and was referred to our psychiatric crisis intervention unit. There he presented himself with sleeping disorders, rumination, anxiety, a lack of drive and recently increasing suicidal ideation. According to the patient, his current depressive episode started over 2 years ago with the loss of his employment and culminated in an acute deterioration a month prior to admission. At our clinic he was diagnosed with a moderate depressive episode (ICD-10 F33.1), reflected by a score of 19 on the 21-item Hamilton Rating Scale (HAMD-21) (24) and by a score of 26 on the patient-rated Beck Depression Inventory (BDI) (25).

**FIGURE 1:**
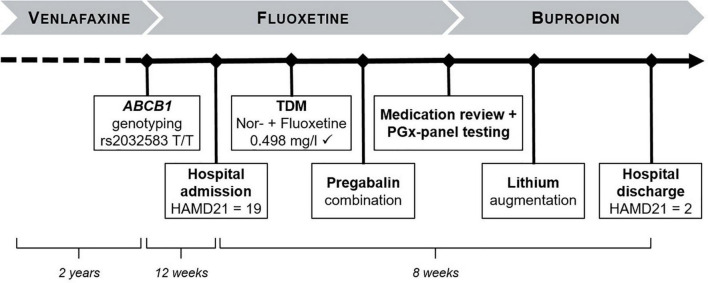
Overview case presentation. TDM, therapeutic drug monitoring; HAMD, Hamilton depression rating scale; PGx, pharmagocenetic.

At clinic entry, the patient was under treatment with a combination of low-dose trimipramine (50 mg/d) for sleep promotion, and fluoxetine (40 mg/d) for depression, which was established 3 months earlier by an outpatient psychiatrist (Figure 1). Before starting this treatment, a long-term treatment with venlafaxine was terminated by the outpatient psychiatrist due to ineffectiveness. His decision to switch to fluoxetine was based on two genetic markers of the *ABCB1* gene, encoding for P-glycoprotein (Table 1), determined in the laboratory of Viollier AG (Allschwil, Switzerland), as recommended by the SGAD (13).

**TABLE 1:** Selected results of the panel pharmacogenotyping and phenotype interpretation.

Gene	Variant (*also tested variants in gene locus*)	Genotype	Diplotype	Predicted phenotype
*ABCB1*	rs2032583, c.2685 + 49T > C, rs2235015, c.497-25G > T *(rs1045642, rs1128503, rs2032582)*	T/T (WT^a^), G/G (WT^a^)	NA^a^	Substance specific function
*CYP2B6*	*(rs8192709, rs28399499, rs3745274)*	WT^a^	*1/*1	Normal function (NM^c^)
*CYP2C9*	*(rs1799853, rs1057910, rs9332131, rs7900194, rs28371685)*	WT^a^	*1/*1	Normal function (NM^c^)
*CYP2C19*	rs4244285 c.681G > A (in *2) *(rs4986893, rs12248560, rs28399504)*	G/A	*1/*2	Reduced function (IM^d^)
*CYP2D6*	*(rs35742686, rs3892097, rs5030655, rs5030867, rs5030865, rs5030656, rs1065852, rs201377835, rs28371706, rs59421388, rs28371725)*	WT^a^	*1/*1	Normal function (NM^c^)
*HTR2A*	rs7997012, c.614-2211T > C, rs9316233, g.47433355C > G *(rs6311, rs6313, rs6314)*	T/T, G/G	NA^b^	Substance specific function

^a^WT, wildtype; ^b^NA, not applicable; ^c^NM, normal metabolizer; ^d^IM, intermediate metabolizer.

Despite a daily dose of 40 mg fluoxetine and steady state trough serum levels within the therapeutic range [fluoxetine + norfluoxetine = 0.498 mg/l, ref. 0.120–0.500 mg/l (26)], the patient did not benefit from treatment with fluoxetine. Therapeutic efficacy did not improve in the in-patient-setting and in combination with pregabalin, which was initiated at the clinic due to restlessness and strain. Due to persisting non-response within the first month of hospitalization, a clinical pharmacist-led medication review including additional pharmacogenetic analysis was initiated. This clinical pharmacy service was part of an observational study approved by the local ethics committee (ClinicalTrials.gov identifier: NCT04154553). The patient gave written informed consent for panel pharmacogenotyping and health data retrieval. A buccal swab was collected to apply the commercial pharmacogenotyping service Stratipharm^®^ offered by humatrix AG (Pfungstadt, Germany). In their laboratory, the polymorphisms are determined by applying real-time PCR using the automated Life Technologies QuantStudio 12 k flex (Thermo Fisher, MA, United States) with the respective optimized and commercially available chemistry. The applied commercial PGx panel test includes genetic variants frequently observed in the European population, including alleles discussed in the CPIC guidelines.^1^ Interpretation of the genotyping results identified the patient as a normal metabolizer (NM, *1 homozygous) for CYP2B6, CYP2C9 and CYP2D6 (Table 1). In addition, the patient’s CYP2C19 phenotype was predicted as intermediate metabolizer (IM, *2 heterozygous) (Table 1). Based on these results and the patient’s history of non-response to venlafaxine, a selective serotonin and norepinephrine reuptake inhibitor (SNRI), and fluoxetine, a selective serotonin reuptake inhibitor (SSRI), a switch to bupropion, a norepinephrine–dopamine reuptake inhibitor, was recommended by the clinical pharmacist. Bupropion is mainly metabolized *via* CYP2B6 and not a substrate of the P-gp transporter (27, 28). After the patient’s medication was switched from fluoxetine to bupropion, a clinical improvement in drive and mood was observable within 1 week. For further improvement and maintenance treatment, an augmentation with lithium was added. Under this combined treatment regimen (Table 2), the patient remitted and was discharged to out-patient care within 4 weeks of treatment change to bupropion, and after a total of 8 weeks of in-patient care. Remission was quantified at discharge with a HAM-D21 score of 2 and a BDI score of 5, compared to 19, respectively, 26 at clinic admission. When followed up 8 weeks after discharge, the patient was still in remission.

**TABLE 2:** Medication at hospital admission vs. at hospital discharge.

Hospital admission		Hospital discharge	
Substance	Schedule	Substance	Schedule
Fluoxetine 20 mg	1-1-0-0	Bupropion 150 mg	1-0-0-0
Trimipramine 100 mg	0-0-0-0.5	Lithium 12 mmol	1-0-1-0
		Pregabalin 75 mg	1-1-0-0
		Pregabalin 100 mg	0-0-1-0
		Colecalciferol 1000 IU	1-0-0-0

### Pharmacogenetic data interpretation and further analysis

Prior to the introduction of bupropion, which eventually proved to be effective, our patient had to endure insufficient antidepressant treatment over the course of more than 2 years. The initial non-response to venlafaxine was attributed to the patients *ABCB1* genotype, with no variation for the polymorphisms rs2032583 and rs2235015. Homozygous carriers of the respective wildtype alleles have been associated with a reduced likelihood of depression remission when treated with antidepressants that are P-gp-substrates. Since venlafaxine is a known P-gp-substrate (15, 29), the treating ambulant psychiatrist decided to switch to the SSRI fluoxetine, a non-relevant P-gp-substrate (30, 31). However, despite these considerations, the patient’s depression deteriorated even further under fluoxetine, necessitating in-patient treatment. There, due to the known involvement of polymorph CYPs in the metabolism of venlafaxine and fluoxetine, further panel pharmacogenotyping was initiated. For venlafaxine, there are PGx-based dosing guidelines available, taking the predicted CYP2D6 phenotype into account (32). Fluoxetine is known to be mainly metabolized *via* the polymorph CYP2D6 and CYP2C9. Although there is no PGx-based dosing guideline available for fluoxetine, genetic variants of *CYP2D6* and *CYP2C9* have been associated with alterations in its pharmacokinetics (33, 34). However, based on the patient’s genetic analysis (Table 1), both CYP2D6 and CYP2C9 are predicted to have normal activity, suggesting that there are no known drug-gene interactions. This is also reflected in the measured nor-/fluoxetine serum levels, which was within the therapeutic reference range at steady state with a common daily dosage of 40 mg.

The non-response to fluoxetine could not be conclusively explained by the assessed pharmacokinetics-related gene variations, including *CYPs* and *ABCB1*. The SERT1 2A (*HTR2A*), which is part of the commercial panel, was also inconspicuous in relation to fluoxetine (12). Therefore, we retrospectively selected the SERT1 (encoded by *SLC6A4*) for further genetic analysis of pharmacodynamic variability. We genotyped for the 5-HTTLPR polymorphism applying the protocol described elsewhere (35) and using gDNA isolated from the patient’s whole blood sample using the QIACube^®^ with the QIAamp^®^ DNA Blood Mini Kit (Qiagen, Hilden, Germany). Herein, the patient presented to be a homozygous carrier of the minor short allele variant in the 5-HTTLPR polymorphism (S/S) of the *SLC6A4*. The 5-HTTLPR S-allele is assumed to cause reduced expression of the SERT1, the target of serotonin reuptake inhibitors including fluoxetine and venlafaxine (19). Several studies associated the 5-HTTLPR major variant, so called L-allele, with an increased likelihood of antidepressant response, especially in Caucasians (22). A recent meta-analysis further specified that the 5-HTTLPR L-allele predicts response specifically to SSRI’s (23). It seems plausible that in the reported case, the present *SLC6A4* variant has indeed affected fluoxetine effectiveness. We hypothesize that this is a relevant reason why the patient clearly benefited from a switch to the noradrenaline and dopamine reuptake inhibitor bupropion, which does not target the genetically affected SERT1.

## Conclusion and outlook

The patient’s *SLC6A4* genotype (S/S) may likely explain why switching to fluoxetine proved ineffective and even led to an acute exacerbation of the depression. The SERT1 is selectively targeted by SSRIs, but its inhibition also contributes to the therapeutic effect of SNRIs and tricyclic antidepressants (36). Consequently, an influence of *SLC6A4* genetic variants on the effect of other antidepressants binding the SERT1 seems plausible. However, the number of studies evaluating the effectiveness of non-SSRI antidepressants in context with *SLC6A4* polymorphisms is still very limited and recent meta-analyses were unable to detect corresponding effects (23, 37). It may be speculated that a pre-emptive approach in PGx testing of the 5-HTTLPR might have significantly reduced the patient’s burden and even avoided hospitalization. Some commercial pharmacogenetic tests already include *SLC6A4* polymorphisms in their panels (38). However, currently there are no recommendations for drug dosing and selection considering polymorphisms in *SLC6A4*. It also seems noteworthy at this point, that besides effectiveness, *SLC6A4* variants have been associated with antidepressant tolerability (39, 40) and even depression susceptibility with *SLC6A4* variation as a potential disease modifying factor (41, 42). Further prospective studies are warranted before genotyping of the SERT1 can be recommended as an additional basis for antidepressant selection. Besides *SLC6A4*, other pharmacodynamically relevant gene variants may gain importance in the near future. Candidate genes under investigation that have been associated with antidepressant efficacy, include genes encoding for the tryptophan hydroxylase (*TPH*), serotonin receptors (*5-HT1A*, *5-HT2A*, *5-HT6*), dopamine receptors (*DRD2*, *DRD4*) and others (18). It is conceivable that pharmacokinetic as well as pharmacodynamic gene variants have a combined effect on the efficacy and tolerability of antidepressants. Therefore, a broader polygenetic approach with panel PGx tests is expected to further gain relevance for a personalized medicine approach in selection and dosing of antidepressants.

## Data availability statement

The original contributions presented in this study are included in the article/Supplementary Material, further inquiries can be directed to the corresponding author/s.

## Ethics statement

The studies involving human participants were reviewed and approved by the Ethikkommission Nordwest- und Zentralschweiz (EKNZ), 4056 Basel, Switzerland. The patients/participants provided their written informed consent to participate in this study. Written informed consent was obtained from the individual(s) for the publication of any potentially identifiable images or data included in this article.

## Author contributions

CS, ML, KH, SA, and HM: conceptualization and study design. CS, RM, ML, and HM: investigation and interpretation of genotyping data. TM and MH: psychiatric clinical assessments. CS: writing—original draft preparation and visualization. RM, TM, MH, ML, SA, KH, and HM: writing—additional content, critical review, and editing. HM: supervision. All authors have read and agreed to the published version of the manuscript.

## Conflict of interest

The authors declare that the research was conducted in the absence of any commercial or financial relationships that could be construed as a potential conflict of interest.

## Publisher’s note

All claims expressed in this article are solely those of the authors and do not necessarily represent those of their affiliated organizations, or those of the publisher, the editors and the reviewers. Any product that may be evaluated in this article, or claim that may be made by its manufacturer, is not guaranteed or endorsed by the publisher.

## Footnotes

1
www.cpicpgx.org
